# Comparison of commercial DNA kits for allergen detection of celery in food matrices

**DOI:** 10.1016/j.heliyon.2024.e36824

**Published:** 2024-08-30

**Authors:** Marleen M. Voorhuijzen-Harink, Bas J. Fronen, Linda Willemsen, Andries Koops, Elise F. Hoek-van den Hil, Nathalie G.E. Smits

**Affiliations:** Wageningen Food Safety Research, Wageningen University and Research, P.O. Box 230, 6700 AE, Wageningen, the Netherlands

## Abstract

For correct allergen risk management by industry, retail and food safety authorities, sensitive and reliable fast allergen detection methods are required, even more when precautionary allergen labelling based on reference doses will be implemented in legislation.

This study aimed to perform a comparative assessment of three commercially available quantitative or qualitative test kits, for DNA analysis of celery in food products. Five product groups, representing different sectors of the AOAC food-matrix triangle, being (plant-based) meat products, snacks, sauces, dried herbs and spices, and smoothies, were identified to potentially contain celery. From each group, blank and incurred (labelled to contain celery) food products were selected, of which the blank food products were additionally spiked with low protein levels of celery prior to qPCR assessment.

Results show that the assessed test kits perform according to their specifications, however, a clear influence of the matrix on the detection ability of celery was observed. In addition, quantification of the amount of celery in the different food products showed to be challenging in all food product groups using the two quantification kits.

## Highlights

• DNA kits detect celery DNA down to 1 ppm spiked protein in five product groups.
• A clear matrix effect was observed.
• Quantitative performance of the test kits was challenging in all food product groups.
• Conversion of DNA results leads to overestimation of the amount of celery.
• Quantification using DNA kits requires optimization for risk management decisions.

## 1 Introduction

Food allergy is an adverse immune response to food and is increasingly prevalent in the past few decades among both adults and children [1]. Physiological responses resulting from food allergy include dermatitis, respiratory problems and gastro intestinal problems. In severe cases the allergic sensitization can lead to anaphylaxis [2]. The CODEX ALIMENTARIUS General Standard for the Labelling of Pre-packaged Foods states eight foods and ingredients that require labelling, namely crustacea, peanuts and soybeans, eggs, fish, cereals containing gluten, milk, tree nuts and sulphite [3]. Countries often adhere to the Codex with individual adaptations per country or region. For example, the European regulation (EU) No 1169/2011 also lists celery, sesame, mustard, lupin and mollusks [4]. In addition to food legislation frameworks, the private Voluntary Incidental Trace Allergen Labelling (VITAL) program is developed to support food manufacturers with a standardized allergen risk assessment process. With the use of this process, food allergens present in a food product as a result of intentional inclusion or cross-contact can be identified, and advice on precautionary labeling is given. To support food manufacturers with data interpretation and decision making the International Life Sciences Institute (ILSI) published a practical guidance document on the application of food allergen quantitative risk assessment [5]. Undeniably, for efficient allergen risk assessment and to supplement the precautionary allergen labelling, sensitive and reliable fast allergen detection methods are required.

To date, DNA-based methods, such as (quantitative) polymerase chain reaction (q)PCR, are increasingly receiving attention with respect to allergen detection, next to the by industry and governmental parties mostly applied ELISA method and the highly selective but time-consuming LC-MS method. Reasons for the increased acceptance of DNA-based methods are their ease-of-use, and the higher stability of DNA versus proteins in thermally processed foods [[6], [7], [8]]. Additionally, DNA-based methods are able to discriminate closely related species such as mustard (a legislated food allergen) and rapeseed (not a legislated food allergen) [9], and celery and carrot [10].

Celery *(Apium graveolens*) was chosen for this comparative assessment study as being one of the ‘substances or products causing allergies or intolerances’ that must be labelled according to EU 1169/2011 [4], and a relevant and difficult to quantify allergen. Celery is cultivated and consumed worldwide in raw, or cooked form, or as a spice, and is associated with mug wort pollen sensitization. For sensitive individuals, even small amounts of celery can already evoke a mild or severe allergic reaction [10]. Moreover, celery is one of the top 14 most common elicitors of confirmed food-induced anaphylaxis [11]. Another reason to select celery is that protein based kits cross-react with other widely consumed species belonging to the *Apiaceae* family [6], for example carrot and parsley, resulting in the current unavailability of ELISA methods [5], and LCMS methods still being under development. In addition, all parts of celery, *i.e.* root, stem, greens and seeds are edible, and consumed raw or processed, making detection and quantification in food matrices even more challenging.

The aim of this study was to evaluate and compare the detection of celery in five food matrix types with commercially available DNA-based test kits.

Five food product or matrix groups, i.e. (plant-based) meat products, snacks, sauces, dried herbs and spices, and smoothies, representing different segments of the AOAC food-matrix triangle were identified to potentially contain celery and were included in this study. Results of this study enable selection of DNA kits suitable for specific needs for celery detection in specific matrixes.

## 2 Material and methods

### 2.1 Characterization of spiking material

No certified reference material was available for spiking celery into the selected food matrices. Therefore, to determine the best spiking material, the four edible parts of celery, to know stem, root (celeriac), greens and seeds, were lyophilized and subsequently subjected to multiple characterization steps focusing on DNA recovery, quality and amplifiability, protein content, and water solubility.

#### 2.1.1 Determination of protein content

All chemicals used were from Merck, Darmstadt, Germany, unless mentioned otherwise.

First, the different parts of the celery plant were freeze-dried and ground, after which the protein content per ground celery part was determined in duplicate using the Kjeldahl method according to Regulation 152/2009 [12] with the following modifications; automated distillation and titration was used with corresponding volumes. Two percent boric acid was used due to practical reasons.

Subsequently the nitrogen content was calculated using the following formula:

$$\%\;nitrogen=\frac{\left(V1-V0\right)\times c\times 14,007\times factor}{m}\times 100$$

V_1_ = hydrochloric acid used in titration (ml)

V_0_ = hydrochloric acid (average) used for blank (ml)

c = concentration hydrochloric acid (in mol/l)

14,007 = mol mass of nitrogen

m = mass of the sample (in mg)

factor = correction factor for the volume delivery of the dosing pump of hydrochloric acid.

To obtain the protein content, the nitrogen content was multiplied by the historically used nitrogen-protein conversion factor of 6.25 [13].

#### 2.1.2 DNA characterization

In short, for DNA characterization, and for each celery part DNA was extracted in five-fold, after which the quantity and quality was assessed. Subsequently, amplifiability was determined by duplicate qPCR analysis, resulting in ten qPCR results per celery part.

#### 2.1.3 DNA extraction of spiking material

DNA was extracted in five-fold from each of the five celery parts using the Maxwell® RSC PureFood GMO and Authentication Kit (Promega, Wisconsin, USA). ∼30 mg of sample material was weighed in a two ml tube, after which one ml of CTAB buffer, 40 μL of proteinase K (20 mg/ml) and five μL of RNase (100 mg/ml) was added, and incubated for 90 min at 65 °C. After incubation, samples were centrifuged for 10 min at 16000×*g*. Then, 300 μL of the supernatant was transferred into a Maxwell cartridge, and samples were further purified in a Maxwell® RSC 48 instrument (Promega, Wisconsin, USA) using the PureFood GMO and authentication method. DNA was eluted in 100 μL elution buffer, and stored at 4 °C for short time storage, or at −20 °C for long time storage.

#### 2.1.4 qPCR

Prior to qPCR analysis, DNA quantity was determined using a spectrophotometer (Nanodrop 1000 instrument, Thermo Fisher Scientific). Samples were diluted to a concentration of 10 ng/μL, of which 5 μL was added to 20 μL of reaction mix containing Taqman Universal Master Mix (Applied Biosystems, UK), and primers and probe (see Table 1). Final primer and probe concentrations were 300 and 200 nmol/L, respectively. Amplification reactions were run on a CFX-96 instrument (Bio-Rad) according to the following protocol: initial steps of 2 min at 50 °C and 10 min at 95 °C, followed by 45 cycles of 15 s at 95 °C and 1 min at 60 °C. Amplification reactions were carried out in duplicate.

**Table 1:** Primer and probe sequence celery qPCR assay.

Primer ID	Sequence (5′-3′)	Reference
Cel-MDH-iF	CGATGAGCGTGTACTGAGTC	[14]
Cel-MDH-iR	AATAGGAACTAACATTAATCATACCAAAC	
Cel-MDH-probe	FAM-AACAGATAACGCTGACTCATCACACCG–BBQ	

#### 2.1.5 Sanger sequencing

In order to taxonomically identify the different celery parts, DNA barcoding was applied using five dedicated plant barcode markers, being rbcL, matK, ITS2, ITS and trnL [15]. The barcode markers were used to amplify short DNA sequences from conserved regions, after which they were compared to the NCBI reference database.

For barcoding the different samples were diluted to a concentration of 10 ng/μL, of which 5 μL was added to 20 μL of reaction mix containing HotstarTaq mastermix (Qiagen, Germany), and primers at a final concentration of 100 nmol/L (see Table 2). Amplification reactions were run on a T100 thermal cycler instrument (Bio-Rad) according to the following protocol: initial denaturation of 15 min at 95 °C, followed by 35 cycles of 30 s at 94 °C, 40 s at 49.5 °C and 1 min at 72 °C.

**Table 2:** Primers using for barcoding.

Assay	Primer ID	Sequence (5′-3′)	Reference
*rbcL*	rbcL a-F	ATGTCACCACAAACAGAGACTAAAGC	[16]
	rbcL a-R	GTAAAATCAAGTCCACCRCG	
*matK*	MatK-1RKIM-f	ACCCAGTCCATCTGGAAATCTTGGTTC	[17]
	MatK-3FKIM-r	CGTACAGTACTTTTGTGTTTACGAG	
*ITS2*	ITS2-S2F	ATGCGATACTTGGTGTGAAT	[18]
	ITS2-S3F	GACGCTTCTCCAGACTACAAT	
*ITS*	ITS4	TCCTCCGCTTATTGATATGC	[[19], [20]]
	ITS5	GGAAGTAAAAGTCGTAACAAGG	
*trnL*	trnL-c	CGAAATCGGTAGACGCTACG	[21]
	trnL-d	GGGGATAGAGGGACTTGAAC	

The resulting amplicons were then purified using QIAquick PCR purification kit (Qiagen, Germany), after which 7.5 μL purified amplicon and 2.5 μL 10 μM primer (forward or reverse) were combined, and sent for Sanger sequencing to Macrogen Europe (Netherlands).

The resulting Sanger sequences were compared to the NCBI reference database using analysis parameters as set by Arulandhu et al. [22].

### 2.2 Description of food products

Five food product groups were identified to potentially contain celery as ingredient: (plant-based) meat products (M), snacks (B), sauces (D), dried herbs and spices (H), and smoothies (S). Per product group, at least two blank (no celery specified on the ingredient list) and four incurred products were selected, except for smoothies as only two incurred products were found to be labelled as containing celery. Specifications of the selected products are given in Table 3.

**Table 3:** Specifications of the used blank and incurred food products.

ID	Food matrix group	Type	Food matrix specifications	% Celery	Tissue/form	Matrix texture
M1	(Plant-based) meat products	Blank	Vegetarian burger	–	–	Solid
M2		Blank	Cooked sausage	–	–	Solid
M3		Incurred	Vegetarian croquette	Not specified	Spice	Solid
M4		Incurred	Vegetable balls	7.2	Not specified	Solid
M5		Incurred	Spicy grill sausage	Not specified	Spice	Solid
M6		Incurred	Baked minced meat	Not specified	Spice	Solid
B1	Snacks	Blank	Beef croquettes	–	–	Solid
B2		Blank	Apple buns	–	–	Solid
B3		Incurred	Oven croquette	Not specified	Spice	Solid
B4		Incurred	Viandel	Not specified	Spice	Solid
B5		Incurred	Vegetable egg roll	Not specified	Spice	Solid
B6		Incurred	Vegetarian bami snack	Not specified	Spice	Solid
D1	Sauces	Blank	Sandwich spread tomato-spring union	–	–	Emulsion
D2		Blank	Pasta sauce	–	–	Emulsion
D3		Incurred	Sandwich spread naturel	Not specified	Spice	Emulsion
D4		Incurred	Egg salad	Not specified	Spice	Emulsion
D5		Incurred	Curry ketchup original	Not specified	Spice	Emulsion
D6		Incurred	Fresh pasta sauce	2.9	Stem	Emulsion
H1	Dried herbs and spices	Blank	Instant tomato soup	–	–	Dried
H2		Blank	Dried Italian herbs	–	–	Dried
H3		Incurred	Herbal soup broth tablets	Not specified	Spice	Dried
H4		Incurred	Instant soup	Not specified	Spice	Dried
H5		Incurred	Herb mixture for minced meat	Not specified	Spice and greens	Dried
H6		Incurred	Herb mixture for stew	1	Root	Dried
S1	Smoothies	Blank	Smoothie mango avocado	–	–	Liquid
S2		Incurred	Smoothie apple-zucchini-celery	Not specified	Not specified	Liquid
S3		Incurred	Tomato juice	Not specified	Not specified	Liquid

### 2.3 Spiking of food products

#### 2.3.1 Units description

The accepted terminology for allergen quantification is *mg total allergenic protein* per *kg food product*. It must be noted that qPCR results are as indicated by the commercial kits given in *mg celery* per *kg food product*.

#### 2.3.2 Spiking procedure

The selected blank materials were spiked with 1, 3 or 10 ppm celery protein (from greens) per kg food product. The selection of the spike level was based on the amount of protein, not the amount of DNA, because the official reporting unit is mg total allergenic protein per kg food product. For spiking the blank matrices, celery greens were diluted to a stock concentration of 100 mg protein/mL using PBS. The stock suspension was further diluted in PBS to reach a final ratio of 0.5 % m/m.

Solid matrices and dried Italian herbs were ground to a fine powder. Diluted stock suspension was added to the fine powder and incubated for 30 min. After incubation water was added and mixed to become a slurry using a hand mixer. The slurry was freeze-dried and ground to a fine powder.

Spiking liquid matrices was performed by adding the total amount of liquid to a beaker with a magnetic stirrer. The diluted stock suspension was pipetted slowly to the liquid matrix and stirred for 10 min.

The emulsion matrices were spiked in a 1 : 1 (m/v) ratio with diluted stock suspension and mixing by using a mortar. A similar amount of emulsion was added repeatedly until the total amount of emulsion was reached. Afterwards the emulsion was mixed for 15 min using a hand mixer.

#### 2.3.3 DNA extraction for homogeneity determination

DNA of the 10 ppm spiked samples was extracted according to the protocol described in paragraph 2.3.1, except that instead of 30 mg, 100 mg input material was used.

Samples were diluted to a concentration of 10 ng/μL, and 5 μL was added to 20 μL of reaction mix containing Taqman Universal Master Mix (Applied Biosystems, UK), primers and probe (see Table 1). Final primer and probe concentrations were 300 and 200 nmol/L. Amplification reactions were run on a CFX-96 instrument (Bio-Rad) according to the following protocol: Initial steps of 2 min at 50 °C and 10 min at 95 °C; followed by 45 cycles of 15 s at 95 °C and 1 min at 60 °C. Amplification reactions were carried out in duplicate.

#### 2.3.4 Homogeneity determination

After spiking the blank materials, homogeneity was determined according to The International Harmonized Protocol for Proficiency Testing of Analytical Laboratories [23] and ISO 13528 [24]. In short, DNA was extracted from 10 replicates of the 10 ppm spiked materials per matrix, after which each replicate was subjected to duplicate qPCR analysis as described in paragraph 2.1.4. Subsequently, using the obtained Cq-values, the between-sample standard deviation (S_S_) of the absolute Cq-values was compared with the standard deviation for proficiency assessment (σ_H_), which was set at 25 % of the grand mean of the homogeneity data [Owen and Gilbert, 2009]. Spiked materials were considered to be homogeneously spiked if S_S_ < 0.3 σ_H_. For matrices D2, M2, H2 and S1 homogeneity could not be determined. For these matrices DNA extraction was repeated after which for matrices D2, M2 and S1 homogeneity data the set requirements were met. For matrix H2 an alternative DNA extraction method, namely the R kit, was used and homogeneity could be established. As the same spiking and homogenization procedure was applied to the 1 and 3 ppm spiked materials, homogeneity was expected, however not confirmed.

### 2.4 Execution of selected kits

In total three commercially available celery qPCR-kits were selected for the comparison study, to know R-Biopharm (R), Biotecon (B) and Generon (G). One kit was specified to be qualitative, and two kits to be quantitative as well. Initially, all kits were subjected to the qualitative performance assessment. Specifications of the kits are shown in Table 4. It is important to note that the LOD and LOQ values are given in ppm celery, contrary to the generally accepted reporting unit for allergens, mg total celery protein per kg food product (see section 2.3.1).

**Table 4:** Specifications of the used DNA extraction and qPCR kits.

ID	Supplier	DNA extraction kit	qPCR kit	Quantitative/qualitative	Recommended standard	LOD	LOQ
R	R-Biopharm	SureFood® PREP Advanced	SureFood® ALLERGEN Celery	Quantitative	Quantard Allergen 40	≤0.4 ppm celery, or 1 celery genome equivalent	1 ppm
B	Biotecon	Foodproof Sample Preparation Kit III	Foodproof® Celery Detection Kit	Quantitative	Allergen RM 800	1 celery genome equivalent, or 0.1 ppm in a celery-spiked rice flour matrix	0.8 ppm
G	Generon	ION FORCE DNA Extractor FAST	SPECIALfinder MC Celery food allergen Real-Time PCR detection kit	Qualitative	SPECIALfinder SpyX	1-10 DNA copies, or 0.5 ppm in a celery-spiked corn flour matrix	–

Per supplier the recommended combination of DNA extraction and qPCR kit was used.

#### 2.4.1 DNA extraction of food matrices

DNA extractions were performed according to the manufacturer's recommendations, including recommended matrix specific adjustments to the protocols.

In short, kit R is a column based DNA extraction method. For all matrices except smoothies and dried Italian herbs, Protocol 2 was used: 150 mg homogenized sample was lysed at 65 °C after which the sample was transferred to a filter column for pre-filtration, binding, purification and first elution with 200 μL elution buffer. Then the filtrate was lysed and bound to a new filter column. After washing and drying of the column, the sample was finally eluted in 50 μL elution buffer and ready-to-use for qPCR. Protocol 1, used for smoothies and dried Italian herbs consisted of only one round of lysis and column purification after which the sample was eluted in 50 μL elution buffer and ready-to-use for qPCR.

Kit B was also a column based method. For all matrices procedure B was used: 200 mg sample was lysed, column purified and eluted in 100 μL elution buffer.

Kit G was column based as well, however larger starting amounts were required and per matrix samples were either filtrated and/or proteinase K treated or not. Details are shown in Table S1 in Supplementary Information (SI).

#### 2.4.2 qPCR analysis

In all cases qPCR reactions were performed according to the manufacturer's manual. In short, kits R and B consisted of initial denaturation, and a two-step (denaturation, and annealing and extension) qPCR with 45, or 50 cycles respectively. Kit G consisted of initial denaturation, and a three step qPCR (denaturation, annealing, and extension) with 35 cycles.

#### 2.4.3 Quantification calculations

Spiked and incurred food products were subjected to quantitative performance assessment for kits R and B. Calculations of the final celery content were performed according to the manufacturer's guidelines. In short, quantification with kit R was based on a five step calibration curve prepared of the supplied reference material and ranged from 50 to 500,000 copies per reaction. In addition, a reference material with known amount of celery was analyzed. The relative quantity of celery in the unknown samples could be calculated using the in the manual specified formula, taking into account the Cq-values of the unknown samples and the reference material, and the slope of the calibration curve. Quantification using kit B was also based on a calibration curve; this by the manufacturer supplied four step calibration curve ranged from 0.8 to 800 ppm celery in a rice-flour matrix per reaction. Subsequently, the relative quantity of celery in a sample was calculated using the linear regression formula generated by the qPCR software (Bio-Rad, CFX manager).

### 2.5 Method acceptance and performance parameters

The quantitative results generated by kits R and B were assessed based on their repeatability, amplification efficiency and linearity. These three parameters were based on guidelines described in the Minimum Information for Publication of Quantitative Real-Time PCR Experiments (MIQE; real-time PCR = qPCR) [25], and guidelines for qPCR analysis of genetically modified organisms (GMOs) [26] and for food foodborne pathogens [27].

#### 2.5.1 Repeatability (RSD_r_)

Results to determine the repeatability (RSD_r_), or intra-assay variance, were as indicated by Marchesi et al. [28] performed under repeatability conditions, *i.e.* using the same method, laboratory, technician, identical samples etc. RSD_r_ refers to the precision and robustness of the assay [25], and when applied to Cq-values, should be below 25 % [[26], [27]]. Here, repeatability of the quantitative kits R and B was determined by amplification of DNA derived from celery greens at levels of 0.5, 1, 3, 5 and 10 mg total allergenic protein per kg food product. These materials were analyzed in duplicate on each qPCR plate resulting in ten values (one plate per matrix group) per level. Subsequently, per level the SD was calculated, divided by the average Cq value, and multiplied by 100. When the resulting percentage did not exceed 25 %, repeatability was considered sufficient.

#### 2.5.2 Amplification efficiency (Ɛ) and linearity (R^2^)

The amplification efficiency (Ɛ) and linearity (R^2^) of the assays was determined based on by the manufacturer recommended calibration curves. The performance requirement for efficiency is set as 80 < Ɛ < 120 % Broeders et al., 2014 in general, or 90 < Ɛ < 110 % for GMO detection [28]. Quantification accuracy of allergens is amongst others, dependent on the set criteria, therefore it was decided to be as accurate as possible and taking Ɛ to be within 90 and 110 % as limit for efficiency acceptance. This corresponds to a slope between −3.1 and −3.6, where −3.32 represents the theoretical perfect slope (amplification efficiency of 100 %).

The R^2^ is the correlation coefficient of a standard curve and its acceptance criterium is generally set as R^2^ ≥ 0.98 Broeders et al., 2014, [27,28].

### 2.6 Statistical analysis

For statistical analysis of the quantitative results, one-way ANOVA with Bonferroni's Multiple Comparison Test with repeated measures in GraphPad Prism4 was used to determine the differences in determined celery quantity per spiking level within one matrix. Results were considered to be statistically different if *P* < 0.05.

## 3 Results and discussion

### 3.1 Characterization spiking material

#### 3.1.1 DNA characteristics

DNA extraction of the four celery parts resulted in recoveries between approximately 50 to 72 μg for seeds and greens respectively, and approximately 60 μg for root and stem, all deemed sufficient for qPCR analysis (Table 5 and S2). The DNA quality represented by the 260/280 nm absorbance ratio was above 1.8 for all four celery parts indicating good quality. However the 260/230 nm absorbance ratio was lower for stem and root compared to greens and seeds, indicating the presence of PCR-inhibiting factors as for instance phenolic compounds. The amplifiability showed to be consistent and efficient with Cq-values between 25.5 and 27.2 for all extracts. On average, seeds resulted in the lowest Cq-values, thereby showing the highest amplification efficiency. Standard deviations (SDs) of the Cq-values of the ten qPCR reactions per part were calculated, and showed to be low, varying from ±0.16 to 0.23 Cq, and comparable between the parts (Table 5).

**Table 5:** Determined characteristics of the four lyophilized celery parts.

Celery tissue	DNA						Protein			Water solubility
	Concentration	Yield	Quality		Amplifiability		N	Content		
	ng/ul	μg	260/280	260/230	avg Cq	SD	% m/m	% m/m^a^	mg/g dry weight	
Stem	199.5	59.8	1.90	1.44	26.81	0.17	1.12	7.0	70 ± 0	Good
Root	188.7	56.6	2.02	1.31	27.06	0.23	1.57	9.8	98 ± 0.9	Good
Greens	239.9	72.0	1.91	1.74	26.50	0.18	3.42	21.4	214 ± 0.9	Good
Seeds	165.4	49.6	1.86	2.01	25.70	0.16	3.08	19.3	193 ± 0.4	Difficult

a N → protein: conversion factor 6.25.

#### 3.1.2 Protein characteristics

Next to DNA assessment, the protein content of the four parts of the celery plant was determined using Kjeldahl. Although being a precise and reproducible method for nitrogen determination, in this study a general conversion factor was used, not taking into account the variation of the nitrogen content of the individual proteins [29]. For the different celery parts the obtained results showed a variability in protein content of 70 mg total protein per g stem, to 214 mg total protein per g greens (Table 5). Finally, the solubility of the celery parts was determined by visual inspection as good solubility and the absence of particulates is a requirement to obtain homogeneously spiked matrices.

Based on the quality and amplifiability of the DNA, and the protein characteristics, greens and seeds were selected for further assessment.

#### 3.1.3 Genetic identification

Greens and seeds were taxonomically identified using DNA barcoding, a technique that can be used for a taxonomic identification. As shown in Table S3, the dedicated plant barcode markers matK, ITS2, ITS and trnL showed resolution down to species level, while rbcL reached the lower genus level resolution. The combined results of the four barcode markers identified both greens and seeds as *Apium graveolens*.

Based on the visual inspection of the spiking material, it was concluded that seeds were less water soluble and therefore not appropriate to be used as spiking material. The three other materials showed good water solubility. Celery greens showed to have the best overall combination of characteristics: high DNA quality and amplifiability, high protein content and good solubility, and were therefore selected as spiking material.

### 3.2 Qualitative assessment spiked and incurred food products

Prior to assessment of the spiked and incurred food products, the homogeneity of the 10 mg total celery protein per kg food product spiked samples was confirmed (Table S4). As the same spiking and homogenization procedure was applied for spiking at 1 and 3 mg total celery protein per kg food product levels, homogeneity of these samples was expected, however not confirmed.

Blank and spiked materials were subjected to qPCR analysis for qualitative assessment using the selected celery detection kits (Table 6).

**Table 6:**
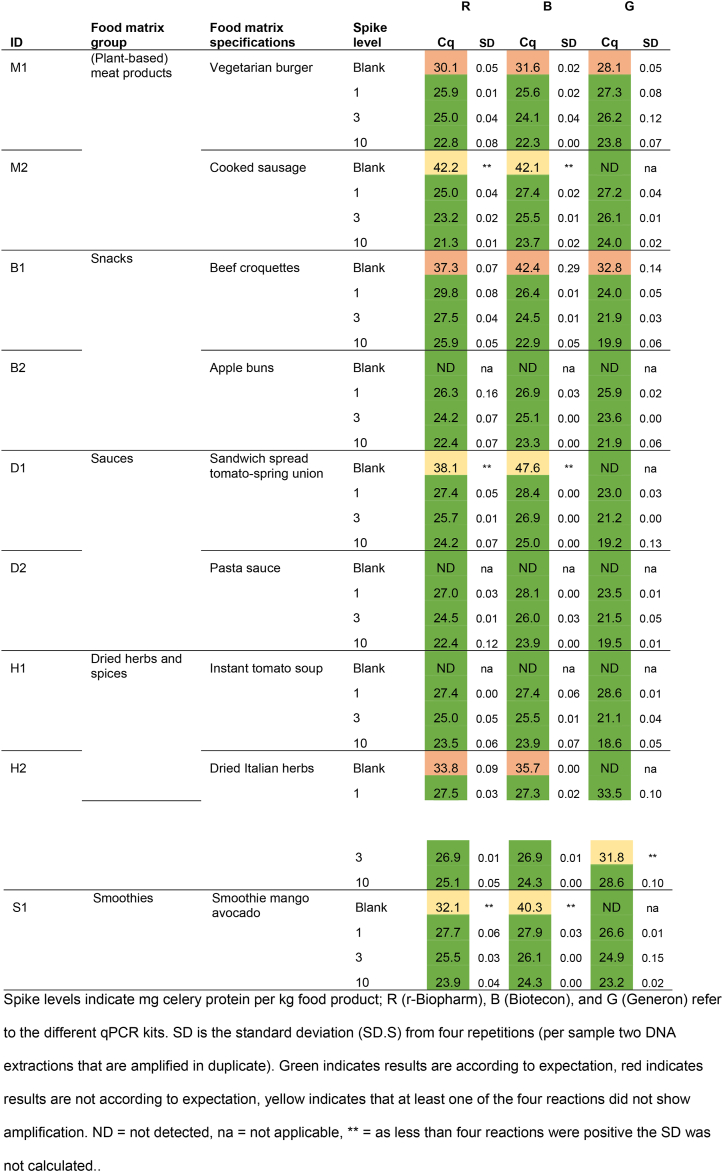
Results of the qualitative assessment of qPCR analysis of blank and spiked celery materials.

The performance of kits R and B showed to be similar (Table 6). Kit G showed small differences in Cq values with kits R and B for four blank samples, namely M2 (cooked sausage), D1 (sandwich spread tomato-spring union), H2 (dried Italian herbs) and S (smoothie mango avocado). Based on the Cq values (Table 6 and S5), it is expected that samples M2 and D1 contain only traces of celery: with kits R and B only one or two out of four reactions resulted in positive, but late amplification (Cq > 38) leading to a ‘possibly detected’ outcome. Kit G (data not shown) did not show amplification in any of the reactions. Sample S1 showed early amplification (Cq ∼ 32) with one of the duplicate kit R DNA extractions, late amplification with one out of four reactions (Cq 40) with kit B, and no amplification with kit G. Lastly, blank sample H2, dried Italian herbs, showed clear and consistent amplification with kits R and B (Cq values between 32 and 36), but no amplification with kit G. Such discrepancies in qPCR results can be explained by: i) difference in amplification efficiency between the kits, ii) difference in corresponding DNA extraction efficiency, iii) a result of possible processing (drying under high temperature) resulting in (partial) degradation of the target DNA, iv) a combination of these. The internal amplification controls (IACs) of these samples, used as indicator for qPCR inhibition (Table S6), showed to generate similar Cq values as the IACs of the positive controls, thereby pointing towards low presence of qPCR inhibitors. However, to determine the origin of the described discrepancies further research is required.

Regarding the spiked samples, kits R, B and G were able to consistently detect DNA equivalent to 1 mg total celery protein per kg food product, thereby confirming the specifications of the kits (Table 4).

#### 3.2.1 Incurred materials

In addition to the blank and spiked materials, per matrix (sub)group two incurred materials, i.e. materials with celery labelled as ingredient, were analyzed. Results are shown in Table 7.

**Table 7:**
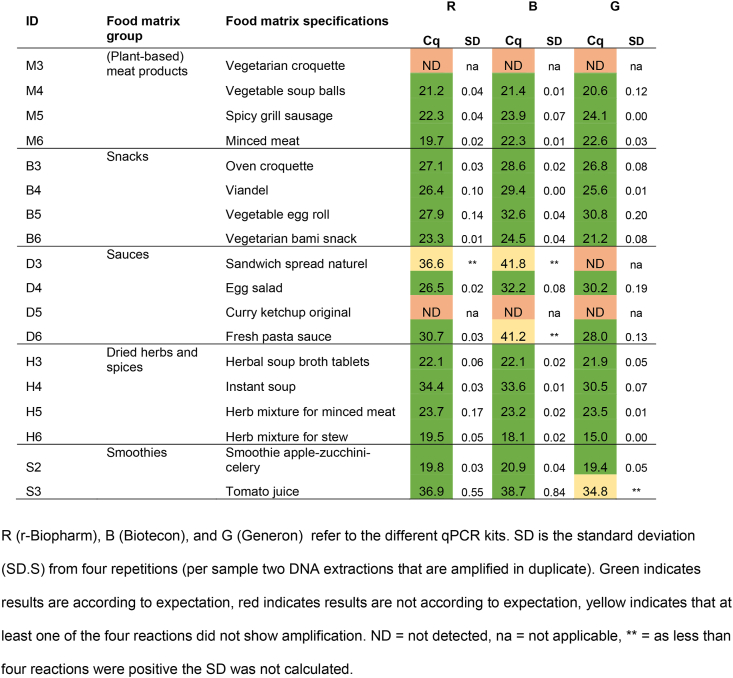
Results of the qualitative assessment of qPCR analysis of incurred materials.

In alignment with the results from the spiked materials, results of the incurred materials (Table 7) indicate similar performance of kits R, B and G regarding qualitative outcomes. In all materials celery was detected with each of the four kits, except in vegetarian croquette (M3), sandwich spread (D1), curry ketchup (D5), and in one duplicate of kit G of tomato juice (S3). Although these materials may contain high amounts of qPCR inhibitory factors as fats, salt, or polyphenols [30,31], and amplification inhibition could be expected, results obtained with the corresponding IACs did not point towards qPCR inhibition. Additionally, these materials showed celery specific Cq values over 36, or no amplification (Table 5 and S6). A perfect qPCR with ideal amplification efficiency theoretically results in Cq 35 when ten target DNA copies are present as input [32], meaning that a Cq value of over 36, indicates even less than one copy of the target in the input material. Therefore, these four incurred materials lacking reproducible amplification are expected to contain merely traces amounts of celery.

### 3.3 Quantitative performance assessment of the test kits

Quantification of allergens by DNA-based methods is currently performed by using an internal DNA standard or a standard curve as used in qPCR [8,33], or digital droplet PCR [34]. Commercially available quantitative allergen test kits mainly rely on quantification using a standard curve, with or without a supplied control or reference material with known amounts of allergenic species Senyuva et al., 2019. In addition, in parallel with the reporting unit in clinical settings, the by FAO/WHO preferred reporting unit is mg of total protein of the allergenic food [35], leading to a required conversion of the obtained results to total protein content for all applied methods [[36], [37]]. Understandably, this is particularly challenging for DNA-based methods, as a DNA quantity cannot be directly linked to a protein concentration.

Kits R and B were subjected to quantitative performance. Quantification with kit R was based on a standard curve in combination with a supplied control with a set amount of celery, kit B used solely a standard curve. Both kits showed correct qualitative performance.

#### 3.3.1 Intra-assay variation

Prior to quantitative assessment of kits R and B, the intra-assay variation, or repeatability, was determined based on the qPCR results obtained by amplification of DNA derived from celery greens at 0.5, 1, 3, 5 and 10 ppm protein input. The calculated RSD_r_ -values of these materials (Table 8) showed to be within the set requirements thereby confirming repeatability.

**Table 8:** Repeatability of kits R and B.

Kit	Spike level (mg protein)	avg Cq ± SD	RSDr (%)
R	0.5	20.35 ± 0.19	0.95
	1	19.04 ± 0.06	0.33
	3	17.45 ± 0.20	1.13
	5	16.48 ± 0.08	0.48
	10	15.31 ± 0.09	0.57
B	0.5	21.51 ± 0.11	0.53
	1	20.14 ± 0.07	0.33
	3	18.39 ± 0.05	0.28
	5	17.64 ± 0.08	0.45
	10	16.43 ± 0.08	0.49

RSDr (relative standard deviation of the repeatability) calculated by (SD/avg Cq)*100, and should be below 25 % above the LOD for quantitative methods.

The obtained calibration curves of each qPCR plate (*i.e.* one per matrix group/kit), showed both kits to meet the set requirements for efficiency (between 90 and 110 %), R^2^ ≥ 0.98, and slope between −3.1 and −3.6 (Table 9).

**Table 9:** Amplification efficiency and linearity of kits R and B.

Kit	Parameter	qPCR plate				
		1	2	3	4	5
R	Ɛ (%)	103.2	98.9	101.0	104.9	105.3
	R^2^	0.999	0.999	0.999	0.998	0.998
	S	−3.2	−3.3	−3.3	−3.2	−3.2
B	Ɛ (%)	98.6	99.8	99.5	99.3	98.1
	R^2^	0.999	0.999	0.999	0.999	0.999
	S	−3.4	−3.3	−3.3	−3.3	−3.4

Ɛ = Efficiency (%), should be between 90 and 110 %; R^2^ = linearity, should be ≥ 0.98; S = Slope, should be between −3.1 and −3.6. R = r-Biopharm, B = Biotecon.

Based on the results of the repeatability, amplification efficiency and linearity, it can be stated that kits R and B meet the set requirements for correct qPCR quantification.

#### 3.3.2 Quantification blank and spiked materials

**Fig. 1:**
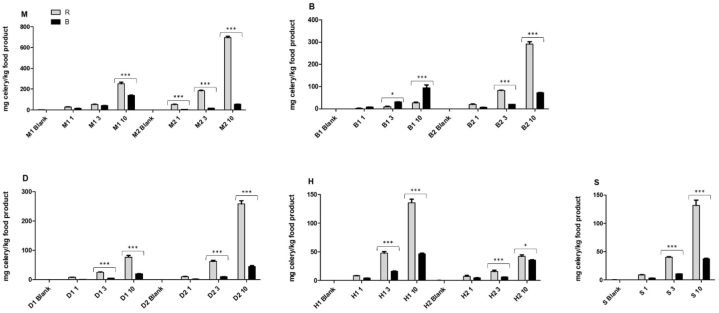
Determined quantities of celery spiked in the different blank materials. Data presented as mean ± SEM. Asterixes (*, *P* < 0.05) are is used to represent a comparative statistically significant result, based on a one-way ANOVA, Bonferroni's Multiple Comparison Test. R = r-Biopharm, B = Biotecon. On the x-axis the different matrices are given (details in Table 3). 1, 3 and 10 represent spiked levels of mg total celery protein per kg food product.

As can be observed from the quantification results of the blank and spiked samples (Fig. 1, and Table S7), quantification of the same materials resulted in large differences between the two kits. For instance, material M2 spiked with 10 mg total celery protein per kg food product resulted in approximately 250 mg celery per kg food product with kit R, while this same material was determined to contain 140 mg celery per kg food product with kit B. Similar results were observed for materials B2 10, D2 10, H1 10, and S 10 mg total celery protein per kg food product. For most materials, except for material B1 at 10 mg total celery protein per kg food product, kit R resulted in higher celery concentrations compared to kit B. Reason for the large differences between the two kits in determined quantities is at least double-barreled. On the one hand there is an effect of the way of determination and calculation of the quantity: solely a standard curve, or a standard curve in combination with a reference material with set amount of celery. On the other hand, and more importantly, there is an effect of the type and age of the tissue used to create the reference/standard material. The type and age of the tissue not only affects the number of cells, and therefore the amount of DNA, but also have an effect on the DNA extractability, and the co-extracted substances [38]. For instance, DNA from young leaves is easily extracted and of good quality, while mature leaves result in lower quality DNA with high concentrations of, amongst others, polyphenols and polysaccharides [39]. Furthermore, seeds or roots are more lignified and DNA extraction will result in more secondary compounds [40]. Therefore, if the unknown, analyzed target is derived from a different tissue, or part, than the tissue/part used as reference in the quantification, this will automatically lead to a deviation in the quantification result. This is especially important for celery as all parts of the plant are edible. While leaves (i.e. greens) were used for spiking the materials, in kit R celery seeds are used as the reference material, for kit B this is not specified. The results obtained by the quantitative assessment underline the importance of the availability of well-characterized reference materials for allergen quantification. Despite that fact, currently reference materials are only limited available.

#### 3.3.3 Quantification incurred materials

**Fig. 2:**
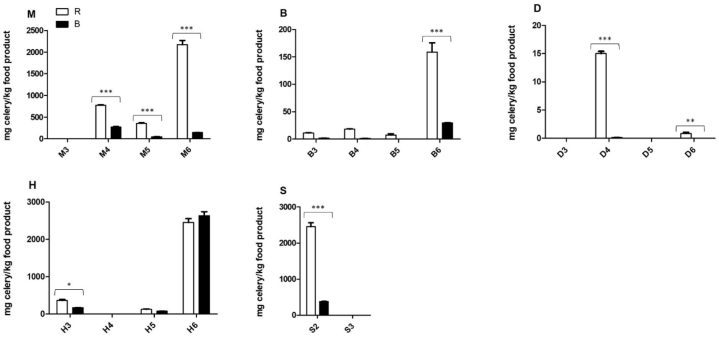
Determined celery quantities in incurred samples. Data presented as mean ± SEM. Asterixes (*, *P* < 0.05) are is used to represent a comparative statistically significant result, based on a One-way ANOVA, Bonferroni's Multiple Comparison Test. R = r-Biopharm, B = Biotecon. On the x-axis the different matrices are given (details in Table 3).

Results of quantification of the incurred materials were comparable to those obtained with the spiked materials, in that way that the determined quantities were generally higher with kit R compared to those obtained with kit B (Fig. 2). For materials M3, D3, H4, and S3 quantification was not possible as no amplification was observed. Table S8 shows the concentrations vs. Cq values (avg) of the incurred materials.

#### 3.3.4 Conversion to protein level

Conversion of the determined DNA-based quantity into a protein-based quantity showed to be challenging. One of the reasons was that DNA quantities were determined using standard curves prepared with supplied materials with known amounts of mg celery per kg food product, and not mg celery *protein* per kg food commodity. Additionally, optimized DNA extraction protocols were applied, rather than protocols for extracting proteins, leading to non-optimal and varying protein levels.

**Fig. 3:**
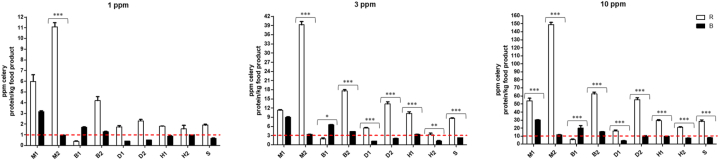
Determined protein quantities of celery spiked in the different blank materials, 1,3 and 10 ppm indicate mg total celery protein per kg food product. N = 4, data presented as mean ± SEM. Asterixes (*, *P* < 0.05) are is used to represent a comparative statistically significant result, based on a One-way ANOVA, Bonferroni's Multiple Comparison Test.

However, taking these considerations into account, the obtained DNA quantities were converted into the preferred mg protein per kg food product reporting unit. First, the protein content of the spiking material was determined to be 21.4 % (m/m) using Kjeldahl (Table 5), then this number was used to convert the output of mg celery DNA per kg food product into mg celery protein per kg food product. Results are shown in Fig. 3 and Table S6.

The outcomes of the conversion showed an overestimation of the amount of total celery protein per kg food product with kit R for the majority of the spiked food materials. Kit B on the other hand showed much lower concentrations of mg total celery protein. As mentioned previously, for kit R celery seeds were used for preparation of the reference material. This might explain the overestimation as seeds, being nutrient storage organs, generally have a higher protein content than leaf materials. However, in this study the protein content of seeds was determined to be comparable to that of greens, i.e. 19.3 versus 21.4 % respectively (Table 5), thereby contradicting the assumption that the overestimation was an effect of difference in tissue type between reference and spiking materials. Kit B did not specify the used tissue type, and therefore no conclusions regarding tissue type could be made.

Although also challenging, the determined protein concentrations were translated into action levels for allergen risk management using the VITAL 3.0 reference dose for celery and the matrix dependent portion sizes as described by Birot et al. [41]. The ED01 from VITAL 3.0 is 0.05 mg total celery protein, regardless of the plant material used (greens, stem, root or seed) in the food product.

As can be concluded from the results in Table 10, protein levels converted out of DNA quantifications cannot be reliable used for risk management. Levels are up to 15 times over- or 0.5 times underestimated depending on matrix and kit.

**Table 10:** Action levels for detection of celery for different types of matrices using the VITAL3.0.

Food matrix group	Food matrix specification (no celery)	Food matrix group description Birot et al.	Portion size (g)	Reference dose total celery protein (mg)	Action level (a)	Nearest spike level (a)	R (a)	B (a)
(Plant-based) meat products	Vegetarian burger	Meal replacements and meat imitates	113	0.05	5.7	10	54.0	30.0
	Cooked sausage	Meat products -mean 105 g such as meat loaf, sausages	126	0.05	6.3	10	148.8	11.5
Snacks	Beef croquettes	Fried/warm snacks	86 (NL), 180 (DK)	0.05	4.3 (NL)	3	2.0	6.6
					9.0 (DK)	10	5.6	20.2
					4.3 (NL)	3	17.6	4.4
					9.0 (DK)	10	62.4	15.4
Sauces	Sandwich spread tomato-spring union	Sauces used as condiments and dessert sauces	30	0.05	1.5	1	1.7	0.4
	Pasta sauce					1	2.3	0.5
Dried herbs and spices	Instant tomato soup	Herbs and spices mixes, bouillon cubes, yeast extract	20	0.05	1.0	1	1.8	0.9
	Dried Italian herbs					1	1.6	1.0
Smoothies	Smoothie mango avocado	Drinks without alcohol (excl. syrup)	483	0.05	24.2	10	28.3	8.0

ED01 reference dose of 0.05 mg total celery protein and portion sizes according to Birot et al. R and B show the calculated concentration (mg protein/kg food product) belonging to the nearest spike level.

NL = the Netherlands, DK = Denmark, R = r-Biopharm, B = Biotecon.

a mg total celery protein per kg food product.

In summary, despite the advantages of DNA-based methods for allergen detection, application poses a number of challenges as well. First, DNA-based methods show the presence of an allergenic species, rather than the presence of an epitope. By using species-specific DNA markers, an indirect proof of the presence of an allergenic compound is obtained. Yet, detection of the allergenic species however suffices according to the food law. Secondly, the matrix i.e. the food commodity in which the potential allergen presence needs to be determined, and food processing not only affect the analytical target itself, but also its extractability, resulting in under- or overestimation of the amount of the allergenic species in the food product [7]. However, as underlined in this study the main challenge for DNA-based methods lies in quantification and subsequent conversion to action levels for allergen risk management.

## 4 Conclusions

This study evaluated the performance of three commercially available quantitative or qualitative DNA-based test kits, for the detection of celery in five food matrices. Per matrix group blank food products, were spiked with three levels of celery greens. In addition, incurred food products were assessed. All kits showed to perform according to their specifications, and were able to detect DNA to the lowest spike level.

Quantification of DNA was challenging, as shown by the results of the two quantitative kits. Results proved to be severely dependent on the complexity of the matrix, shown by the variability between the two kits in determined celery quantities in the different food products. In addition, as a DNA concentration cannot be directly associated with a protein concentration, conversion of the DNA quantity to the reporting unit that is preferred by FAO/WHO (mg of total protein of the allergenic food) is required. Moreover, the obtained analytical results need to be converted to the allergen concentration in the food product [5]. Results here show that conversion of the DNA concentration to mg total celery protein per kg food product in the vast majority leads to an overestimation of the amount of celery. Although overestimation is regarded as being on the safe side, as action levels are met even when the allergen is present at a lower level, more accurate quantification is required. Therefore, to obtain more reliable results for risk management decisions quantification using DNA-based methods and the application of corresponding conversion factors must be further optimized.

Lastly, comparative evaluation with different strategies, e.g. ELISA or LC-MS would be desirable for the detection of celery, however these are currently not available due to cross-reaction with other species [5], or are under development.

## Data availability statement

No data was used for the research described in the article.

## Funding

This project was financially supported by the Dutch Topsector Agri&Food in project LWV19252.

## CRediT authorship contribution statement

**Marleen M. Voorhuijzen-Harink:** Writing – original draft, Methodology, Data curation, Conceptualization. **Bas J. Fronen:** Methodology. **Linda Willemsen:** Methodology. **Andries Koops:** Writing – review & editing, Funding acquisition. **Elise F. Hoek-van den Hil:** Writing – review & editing, Project administration, Funding acquisition, Conceptualization. **Nathalie G.E. Smits:** Writing – review & editing, Project administration, Methodology, Funding acquisition, Conceptualization.

## Declaration of competing interest

The authors declare that they have no known competing financial interests or personal relationships that could have appeared to influence the work reported in this paper.
