# Supplementary material for: Comparison of commercial DNA kits for allergen detection of celery in food matrices
Source: Heliyon. 2024 Aug 30;10(17):e36824. doi: 10.1016/j.heliyon.2024.e36824 (PMC11419853; doi:10.1016/j.heliyon.2024.e36824)
Supplement: Multimedia component 1 [file mmc1.docx]

**Supplementary Information**

**Comparison of commercial DNA kits for allergen detection of celery in food matrices**

Marleen M. Voorhuijzen-Harink^a^
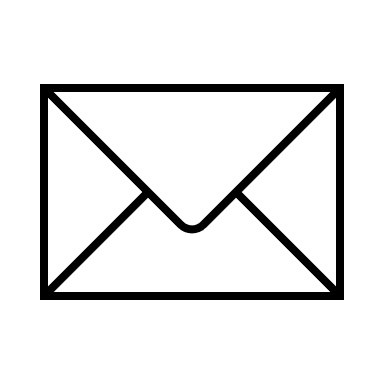
, Bas J. Fronen^a^, Linda Willemsen^a^, Andries Koops^a^, Elise F. Hoek-van den Hil^a^, Nathalie G.E. Smits^a^

^a^ Wageningen Food Safety Research, Wageningen University and Research, P.O. Box 230, 6700 AE Wageningen, The Netherlands.


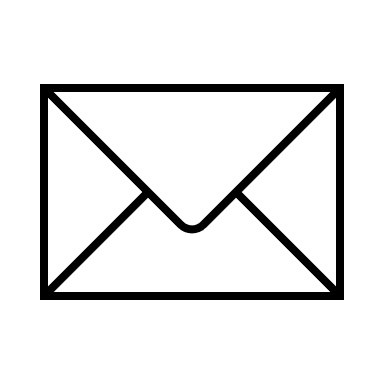
 Corresponding author: [Marleen.Voorhuijzen](mailto:Nathalie.Smits@wur.nl)@wur.nl

Contents

[Table S2. Results characterization spiking material. 3](#_Toc173996357)

[Table S3. Sanger barcoding results of celery greens and seeds. 4](#_Toc173996358)

[Table S4. Results homogeneity determination 10 ppm spiked materials. 4](#_Toc173996359)

[Table S5. Cq-values spiked and incurred materials. 5](#_Toc173996360)

[Table S6. Cq-values internal amplification controls spiked and incurred materials. 6](#_Toc173996361)

[Table S7. Concentrations vs Cq values (avg) of the blank and spiked materials. 6](#_Toc173996362)

[Table S8. Concentrations vs Cq values (average) of the incurred materials. 7](#_Toc173996363)

Tables

Table S1. DNA extraction specifications kit G.


### Table S2. Results characterization spiking material.


*N --> protein: conversion factor 6.25

### Table S3. Sanger barcoding results of celery greens and seeds.

### Table S4. Results homogeneity determination 10 ppm spiked materials.

### Table S5. Cq-values spiked and incurred materials.


N/A = not amplified. R = r-Biopharm, B = Biotecon, G = Generon.

### Table S6. Cq-values internal amplification controls spiked and incurred materials.


N/A = not amplified. R = r-Biopharm, B = Biotecon, G = Generon.

### Table S7. Concentrations vs Cq values (avg) of the blank and spiked materials.


ND = not detected, NQ = not quantifiable, * = at least one of the four amplifications is not detected.
R = r-Biopharm, B = Biotecon.

### Table S8. Concentrations vs Cq values (average) of the incurred materials.


ND = not detected, NQ = not quantifiable, * = at least one of the four amplifications is not detected.
R = r-Biopharm, B = Biotecon.

Figures

Figure S1. Comparison of the determined celery quantities (mg celery/kg food product) per spiked level, obtained with the two quantitative kits. R = r-Biopharm, B = Biotecon. On the x-axis the different matrices are given (details in Table 1).
